# Evaluation of dietary habits during pregnancy

**DOI:** 10.4274/tjod.79923

**Published:** 2015-06-15

**Authors:** Nihan Şenol Eren, İrfan Şencan, Hilal Aksoy, Esra Meltem Koç, İsmail Kasım, Rabia Kahveci, Gülhan Samur, Adem Özkara

**Affiliations:** 1 Şehit Kamil Family Health Center, Gaziantep, Turkey; 2 Ankara Numune Training and Research Hospital, Clinic of Family Medicine, Ankara, Turkey; 3 Community Health Center of Etimesgut, Ankara, Turkey; 4 Community Health Center of Mamak, Ankara, Turkey; 5 Hacettepe University Faculty of Medicine, Department of Nutrition and Dietetics, Ankara, Turkey; 6 Hitit University Faculty of Medicine, Department of Family Medicine, Çorum, Turkey

**Keywords:** Pregnancy, Nutrition, Awareness

## Abstract

**Objective:**

Pregnancy is a special period of increased nutritional needs during which conscious nutritional support is required. Insufficient and imbalanced nutrition in this period of life causes serious conditions that affect both child and mother. This study aimed to evaluate the relationship between pregnancy and nutrition/nutritional habits during pregnancy.

**Materials and Methods:**

In this descriptive study, a questionnaire was conducted on a voluntary basis to pregnant women who were admitted to the Pregnancy Outpatient Clinic of Obstetrics and Gynecology Department at Ankara Numune Training and Research Hospital. Questions about general information, pregnancy-related information, thoughts and knowledge about breastfeeding, nutritional habits, and meal frequency were asked to pregnant women. Three hundred fourteen questionnaires were assessed in the study. SPSS for Windows Version 16.0 and MS-Excel 2007 were used for statistical evaluations. P<0.05 was accepted as statistical significance.

**Results:**

There was a statistically significant relationship between pre-pregnancy body mass index (BMI) and number of pregnancies; level of education and income levels; number of children and history of caesarian section as an additional problem within previous pregnancies. The change of nutritional habits during pregnancy was examined; we found that consumption of fruits (51%) and vegetables (40.8%) increased the most, while intake of tea (26.1%) and redmeat (21%) mainly decreased during pregnancy. It was found that during pregnancy 20.4% of pregnant women had never consumed fish, 13.1% abstained from red meat, and 12.4% excluded white meat from their diet.

**Conclusions:**

We believe that this study will help to raise awareness about adequate and balanced nutrition during pregnancy and to define special nutritional recommendations.

## INTRODUCTION

Adequate consumption of required nutrients according to an individual’s age, sex, and physiologic environment, generates the main purpose of nutrition^(1)^. The World Health Organization (WHO) admits that the proccess of malnutrition starts in the womb and continues until death. Insufficient and unbalanced nutrition problems especially occur in developing countries at every stage of life and can cause serious health problems if precautions are not taken^(2)^.

Nutrition in various stages between food suply and intake are affected by deficiencies and irregularities in the technology of food production and distribution, socioeconomic and cultural factors, lack of purchasing power, family structure, various customs and traditions, environment, and education^(1)^.

Nutritional needs increase and nutrition should be consciously considered during pregnancy. Women need to be healthy and have a regular diet before getting pregnant, to facilitate pregnancy, to carry their child until to term, to have her child at normal weight and time, and to breastfeed^(3,4)^.

Insufficient and unbalanced nutrition during pregnancy for babies may result in premature birth, stillbirth, death in the first months after birth, miscarriage, physical abnormalities, and mental retardation; for mothers, death, anemia, osteomalacia, tooth decay, thinness or fatness, iodine deficiency, gestational diabetes (GD), eclampsia, and pre-eclampsia^(5)^.

The main purpose in this study was to research the effects of diet on pregnancy and to investigate the awareness of proper nutrition through the evaluation of pregnant womens’ opinions on dietary habits during pregnancy.

## MATERIALS AND METHODS

This sectional survey study was conducted in the Pregnancy Polyclinic of the Obstetrics and Gynecology Clinic at Ankara Numune Training and Research Hospital between March 2011, and November 2011. Approval for the study was obtained from Ankara Numune Training and Research Hospital Ethics Committee.

In this study, a detailed survey that evaluated sociodemographic characteristics, pregnancy-related information, thoughts on breastfeeding, dietary habits and attitudes about food consumption frequency was conducted to pregnant women after their oral and written approval was given. Each woman was informed how to complete the survey. Eight of the participants were illiterate and were helped by their relatives and researchers.

Information was also collected on the womens’ Hb, Hct, and blood pressure level, which were checked routinely at our pregnancy polyclinic.

Information obtained from the subjects were digitized and quantified. Clean data was provided with cross-checks with necessary error checks and correction. Suitability for continuous variables (eg age, education level, parity, health insurance) to normal distribution was examined graphically using the Shapiro-Wilk test. Descriptive statistics for continuous variables are shown with average ± standard deviation or median (IQR-interquartile range) related to normal distribution. For the display of categorical and classified variables, cross tables with number and percent were used. Student’s T-test or Mann-Whitney U-test were used when comparing continuous variables according to test subjects. Differences among the individual categorical variables were evaluated using Chi-square, Chi-square likelihood ratio, or Fisher’s exact Chi-square with preparing cross tables.

SPSS for Windows version 16.0 (SPSS Inc. Chicago, IL., USA) and MS-Excel 2007 were used for all the statistical analyses and calculations. P<0.05 was accepted as statistical significance.

## RESULTS

Three hundred twenty eight pregnant women participated in the study. Fourteen of which were excluded for failing to complete the questionnaire properly. A total of 314 questionnaires were evaluated.

### Socio-demographic characteristics of the pregnant women

The women in the study were aged between of 16 and 45 years. The average age was 26.50±7 years. Two hundred fifty-four of the women were aged between 20 and 34 years (80.9%). The participants’ sociodemographic characteristics are given in Table 1.

Two hundred sixty four (84.1%) were housewives. The remaining 50 (15.9%) were employed.

There was a statistically significant correlation between income and education level in the results of the analyses (p=0.024). There was no correlation found between income level and number of children when evaluated statistically (p=0.643).

### Pregnancy-related information

Eighty-seven (27.7%) of the pregnant women were in the first trimester, 113 (36%) in the second, and 114 (36.3%) in the third.

The womens’ clinical signs such as weight, height, body mass index (BMI), and blood pressure are given in Table 2.

The women in the study were weighed, weights ranged from 44-101 kg during pregnancy. The range of weight changes during pregnancy was found to be 6 kg lost and 26 kg gained. The median weight change was 5.0±9.0 kg. There was no correlation found between employment status and weight gain during pregnancy (p=0.335). Participants who had been pregnant before were evaluated according to their previous pregnancy. The number of women who saw an increase in weight up to 5 kg was 43 (13.7%), between 6-10 kg was 62 (19.8%). Fourteen (4.5%) women gained more than 20 kg.

There was a statisticaly significant correlation found between BMI and number of pregnancies (r=0.176, p=0.002). However, this relation was regarded as poor. The strength of this relation was 17.6%.

Hypertension was detected (140/110 mmHg) in one woman who is in the 2^nd^ trimester; the woman had also had hypertension in her previous pregnancy.

Anemia was found in 5 (5.7%) out of 87 women in the 1^st^ trimester, 13 of 113 in the 2^nd^ trimester, and 19 (11.8%) of 114 in 3^rd^ trimester. Anemia criteria was evaluated as Hb 11.0 mg/dL in the 1^st^ trimester, 10.5 mg/dL in the 2^nd^ trimester, and below 11 mg/dL in the 3^rd^ trimester^(6)^.

The number of women who had consulted a doctor prior to pregnancy was 95 (30.3%). The number of consultations was once in 87 (27.7%) women, and twice or more in 8 (2.6%).

Ninety-two (30.3%) of the participants stated that they took folic acid, vitamin B12, and iron before pregnancy.

Three hundred twelve (99.3%) of the women stated that their doctors had recommended that they should take vitamins after becoming pregnant. One woman (0.3%) stated that she took vitamins on receiving advice from a pharmacist, and another (0.3%) received advice from a neighbour.

For 100 (31.8%) of the women, it was their first pregnancy. One hundred twenty-two (38.9%) of the women had no children, and 20 (6.3%) had 3 or more children.

Some 103 (32.8%) of the women had had one or more problems in their previous pregnancies related with pregnancy or birth. Fift-eight (18.5%) had had a c-section, 40 (12.7%) had miscarried. There was no correlation between not gaining weight and presence of health problems (p=0.380). There was no statistical difference between education level and presence additional disease related to pregnancy or birth (p=0.29). There was a statistical difference between number of children and c-sections that occured in previous pregnancy or birth (p<0.001). The number of c-sections reduced as the number of children increased. There was no statistical correlation between income level and additional diseases (p=0.703). There was no relation between age and additional diseases (p=0.570). A relation was detected statistically between BMI before pregnancy and health problems during previous pregnancy and birth (p=0.005). There was a statistical relation between those who had seen a dietician and not in the aspect of additional disease existence (p=0.029). No relation was found between those shorter than 150 cm and existence of additional disease (p=0.205).

When the duration between 2 pregnancies was evaluated, the number of women who had a less than 2-year gap between their 2 pregnancies was 91 (28.9%); 6 (4.1%) women had a gap of more than 10 years.

One hundred ninety-five (62.1%) of the womens’ pregnancies were planned pregnancy, 119 (37.9%) were unplanned. There was no statistical relation between planned pregnancy and anemia (p=0.395).

Three hundred ten (98.7%) women were under the supervison of a doctor. Four (1.3%) women were not being supervised by a doctor during pregnancy because of various reasons.

Twenty (6.4%) of the women had seen a dietician during their pregnancy; however, 33 (10.5%) had a special diet program. Of those, 14 (4.5%) of the women said that they had a special diet program for specific reasons from their doctors, 14 (4.5%) from had diets from their dietician, 4 (1.3%) from their nurses. One (0.3%) woman said that she had taken a program from her environment (tv, friend, neighbor).

For preexisting medical conditions, 71 (22.6%) of the women had no additional disease before pregnancy; 15 (4.8%) had hypertension; 8 (2.5%) had diabetes mellitus, 8 (2.5%) had hyperlipidemia; 25 (7.9%) had thyroid diseases; 3 (0.9%) had preeclampsia; 2 (0.6%) had GD. Nine women stated that they had a preexisting condition but did not specify it in the questionnaire.

The number of women who used medicine during pregnancy because of their preexisting condition was 41 (13.1%).

There were 44 (14%) women who smoked during current pregnancy. There was no statistical relation between education level and smoking (p=0.163).

Twenty-five (8%) of the women smoked 1-5 cigarettes per day, 13 (4.1%) smoked between 6-10, and 6 (1.9%) smoked 11 and more cigarettes.

### Thoughts on breastfeeding

Three hundred twelve (99.4%) of the particpants had planned to breastfeed their baby; 281 (89.5%) said that they knew for how long they needed to only breastfeed their baby. The number of the women who thought they were only going to breastfeed their baby for 6 months was 208 (66.2%); 224 (71.3%) were considering switching to additional food after 6 months. One hundred sixty-eight (53.5%) women were planning to breastfeed for more than 18 months. According to the answers provided by the women, 86 (26.4%) women had breastfed their previous child for 6 months, and 48 (15.3%) had breastfed for 18 months or more.

### Changes in diet during pregnancy

When the change of main food groups consumed during pregnancy were evaluated, the most increased food groups consumed were fruits (51%) and vegetables (40.8%); the most decreased food group consumed were tea (26.1%) and red meat (21%). White meat and fish consumption decreased 20.1% and 27.7%, respectively. When the food consumption was evaluated, the most frequently consumed foods were bread (85.3%), oil (66.2%), cheese (67.5%), and milk-yogurt (57.9%). Some 38.5% of the women were consuming eggs daily. Foods that were never consumed during pregnancy were molasses (41.4%), butter (41.1%), meat products like salami-pepperoni (40.8), fish (20.4%), red meat (13.1%), and white meat (12.4%).

## DISCUSSION

Maternal health markers that are accepted by the Pregnancy surveillance system (PNSS) as important factors that can be affected by diet are BMI before pregnancy, maternal weight increase, anemia, GD, and hypertension during pregnancy. In addition, the number of pregnancies, time between two pregnancies, age of mother, present additional diseases, drugs, income level, education level, health insurance, smoking-drinking habits also affect maternal health directly or indirectly^(6)^.

Previous researche revealed that the highest rate giving birth was in women aged 20-24 years. In the Turkish Demographic and Health Research (TDHR) in 2008, it appeared that for first time the age of women who gave birth the most had moved to the 25-29 years age group. This result shows that specific fertility patterns have changed and births were being postponed^(7,8)^. In our study, 31.5% of the women were aged 20-24 years and 32.5% were aged 25-29 years.

As education level increases, so does welfare. According to the data of TDHR 2008, 48% of the women had low welfare levels and did not complete the first stage of elementary school; the highest welfare was in only 2%^(7)^. Of the women who participated in this research, 2.5% were illiterate, all of whom had 1000 TL or less monthly income. Of the women who had monthly incomes below 1000 TL, 67.2% had elementary and lower education levels. There was a statistical correlation between education and income level. According to our research, as education level increases, income and therefore welfare levels of families also increases.

When considering pregnant women’s height, risks in birth are predictable. Short women have a small and narrow pelvis; therefore, height is a useful parameter in predicting birth risks and as such is important for health of mother and child. Women who are 140-150 cm are considered under threat of potentially risky births; according to the results of TDHR 2008, the average height for mothers was 157 cm and was 158.1 cm according to TDHR 2013 results. Ten percent of mothers are shorter than 150 cm^(6,7)^. Of the women in our study, 5% were in the risk group (<15 cm); no relation was found between short stature and additional diseases related to pregnancy or birth.

According to the results of TDHR 2013, the average BMI for women aged 15-49 is 26.7. According to TDHR 2008, 2% of women’s BMI was lower than 18.5, 24% had a BMI higher than 30. According to TDHR 2013, 3.6% of women’s BMI was below 18.5 and 27% had a BMI of 30 and higher^(7,8)^. The women in our study were asked about their weight prior to pregnancy to obtain their dietary habits. The percentage (4.8%) of women in this study who had a BMI below 18.5 before pregnancy was higher than the TDHR 2008 and TDHR 2013 values. Some 31.8% of the women in this study were having their first pregnancy, which may explain why the BMI values were lower than Turkey’s average. A statistically significant relationship was found between BMI before pregnancy and number of pregnancies. Therefore, if women are informed that each pregnancy makes permanent changes in their weight, potential obesity after pregnancy should be prevented. With preventing obesity, the progress of many chronic diseases such as diabetes and hypertension can be delayed and may be prevented. To prevent permanent obesity after pregnancy, behavioral changes such as diet and exercise should be introduced to mothers during pregnancy and after giving birthgiving. A significant difference was found when the women’s BMI before pregnancy and the presence of additional disease related to birth or previous pregnancies were compared statistically; we may conclude that the correlation between obesity and additional disease may occur at any age.

The women’s BMI was calculated and weight gain was evaluated in our study. The women in the 1^st^ trimester gained 1.32±2.84 kg, 4.05±4.27 kg in the 2^nd^ trimester, and 10.94±5.31 kg in the 3^rd^ trimester. Erdem studied 95 pregnant women aged between 15-35 years who presented to Ankara Public Hospital Obstetrics and Gynecology Clinic, the women gained 12.95±6.82 kg during pregnancy, 1.2±2.7 kg in the 1^st^ trimester, 3.6±3.3 in the 2^nd^ trimester, and 5.3±3.2 kg in the 3^rd^ trimester^(9)^. When we compared the two studies, weight gain was found to be similar because weight gain was also calculated for each trimester separately in Erdem’s study. It appears that there are pregnant women who do not reach the advised weight and others gain excess weight. This situation can cause fetal malnutrition and negatively impacts on mother’s health. Therefore, increasing awareness about nutrition and referral to a dietician if necessary might be appropriate for women who are pregnant.

Mineral complex and iron+folic acid were compared in Cochrane literature. With use of both, miscarriage and anemia rate were found to be low but no difference was found between the two groups^(10,11,12)^. Almost all of the women in this study were using vitamins during their pregnancy. Almost all (99.3%) had been advised to use vitamins by their doctors. This situation is related to pregnant women being under medical supervision.

In a similar study by Irge et al., it was found that 46.5% of the pregnant women took vitamins or minerals and 20.3% used iron and vitamin supplemnts together^(13)^. Kılıç et al. found that 67% of the pregnant women in their study took vitamins and minerals^(14)^. Sözeri et al. found that most of their pregnant women had regular health checks, regularly use their prescribed medicines, and took care about their sleep and diet^(15)^. The percentage of women who did not use preconceptional folic acid was 70.7%. Therefore, it appears that women should be informed about not using folic acid before pregnancy. Women should be also informed about diet during pregnancy but use of multivitamin-minerals should not be insisted upon.

In a study of 100 pregnant women age between 16-40 years in the Gülveren district of Ankara by Sağlam and Baysal, the percentage of the women with anemia (Hb value below 11 mg/dL) was 52% In our study the percentage of the women with anemia was lower^(16)^. But Hb value below 10,5 mg/dL was considered as anemia. This could be one of the reasons of the difference between studies. Also the difference in our study is likely explained by the introduction of the iron support program for women by the Ministry of Health in 2005, which resulted in a widespead increase in use of iron preparations.

Of the women, 7.4% were having a planned pregnancy; 5.7% of those who were not having a planned pregnancy had anemia. When it was evaluated statistically, the non correlation between planned pregnancy and anemia suggests that wider support programs should be provided to explain the importance of using folic acid and iron to women who are planning a pregnancy.

In Turkey, the long and the median birth interval were 44 and 45 months in TDHR 2008 and in 2013, respectively; both were very similar. Long-term breastfeeding and temporary infertility after birth contribute to long birth intervals to be at relatively high levels in Turkey. According to TDHR 2008, three-fifths of pregnancies occured at least 3 years after the previous pregnancy, a little more than one-fifth occured between 24-35 months; in TDHR 2013, two-thirds of pregnancies occured at least 3 years after the previous pregnancy and nearly 19% occured beween 24-35 months^(7,8)^. In the present study, the percentage of women whose 2 pregnancies were less than 24 months apart was 28.9%.

Smoking can cause low birth weight by negatively affecting intrauterine growth. It is believed that smoking has a negative impact on breast milk volume of mothers who smoke, which may render it inadequate to support the energy requirements of their babies^(17)^. Andreas and Day reported that 15-20% of pregnant women smoke, and that smoking was responsible for 15% of the preterm babies and 20-30% of the babies with low birth weight in their study^(18)^. In the study of 1583 pregnant women by England et al. the effect of reducing smoking by 50% and more was only 32 grams on birth weight, the difference was not statistically significant^(19)^. The authors concluded that when smoking increases in the third trimester, birth weight is clearly decreased, but pregnant women need to decrease daily cigarette consumption to below 8 because birth weight stayed stable after 8 cigarettes. In the 10-year study of Chang et al. of 1120 adolescent pregnant girls aged 17 years and under, a statistically significant relationship was found between inadequate weight gain during pregnancy, low BMI, and low birth weight caused by smoking^(20)^. Fourteen perecent of the women in our study continued smoking. Fifty-six percent of the smokers had elementary education, 34.1% went to high school, and 4.5% were university graduates. No relation was found between education level and smoking in our study. When the study was compared to Turkey in general, the rate of women smoked during pregnancy was higher. This difference may be due to the fact theat participants in this study lived in urban areas. In addition, it can be considered the women who presented to our hospital were not sufficiently informed about this subject. It would be appropriate to educate pregnant women and encourage them to quit smoking though identifiying smokers who present to our hospital or any hospital in Turkey.

To evaluate the nutritional status of the women who participated in our study, we evaluated dietary habits and changes made regarding the amount of essential nutrients consumed prior to pregnancy and consumption frequency of these food groups. When the change of essential food groups being consumed during pregnancy was compared with the consumption before pregnancy, the percentage of women who never consumed fish during pregnancy was 20.4%. It is very important to consume fish and other sea food that contain omega fatty acids, especially during pregnancy^(5)^. The percentage of women who had consumed no red or white meat (chicken, turkey) were 13.1% and 12.4%, respectively. The rate of women who consumed insufficient meat might have been be higher because we did not specifically enquire about the amount food consumed. It is not possible to reach a conclusion about why the women chose not to consume certain foods. These are important to issues to investigate in subsequent studies.

Breastfeeding is quite common in Turkey. Deveci et al. conducted a study in two health centers in urban and semi-urban regions of Manisa, with the mothers who had a child aged 0-24 months; 97.4% of the babies had been given breast milk^(21)^. In another study by Yıldız et al., all mothers breastfed their babies for a period and 88.4% continued breastfeeding their child after 1 year^(22)^. In our study, the percentage of the women who only gave breast milk to their previous child for 6 months was 40.1%. Although breastfeeding is common in Turkey, the rate of feeding only with breast milk was 42% in TDHR 2008, whereas in TDHR 2013 it decreased to 30%; therefore, feeding only with breast milk may not be as common as is widely suggested^(8)^. There were similarities between the rate of feeding only with breast milk in our study and TDHR 2008 data. All this ratio is insufficient and the importance of a 6-month period of feeding with breast milk alone should be kept on the agenda. Mothers should be encouraged to breastfeed.

Nutrition is the most important factor that needs to be paid attention to in order to live a healthy life. The effects of nutrition in humans begin in the womb. Therefore, women who are pregnant should be more aware and consume an adequate and balanced diet for themselves and their babies in this period when the need for calories, protein, vitamins, minerals, liquids, and basic and trace elements are increased, such that their baby may be born healthy. Our study shows that many women in pregnancy do not gain enough weight or gain excess weight; do not use preconceptional folic acid; and smoke cigarettes. Women in pregnancy feed insufficently and do not consume some essential nutrients. Also, many are reluctant to breastfeed.

We believe that our study will raise awareness about adequate and balanced nutrition during pregnancy and help physicians to learn topics that need to be paid particular attention.

To raise future generations with higher awareness of the importance of nutrition during pregnancy, more studies should be performed on this issue and necessary health policies should be introduced.

## Figures and Tables

**Table 1:**
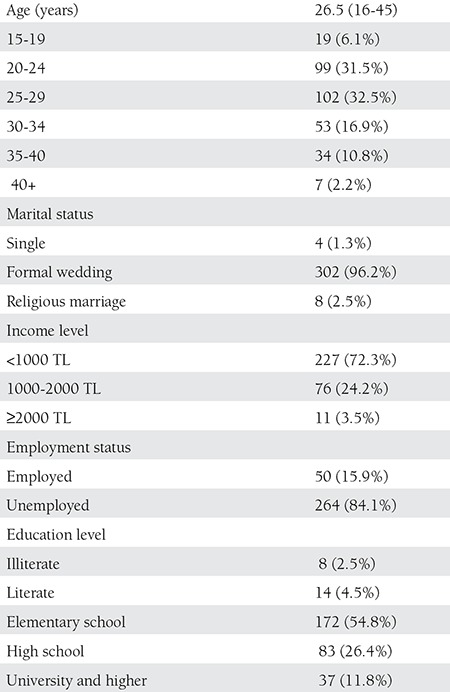
Sociodemographic feautures of the women

**Table 2:**
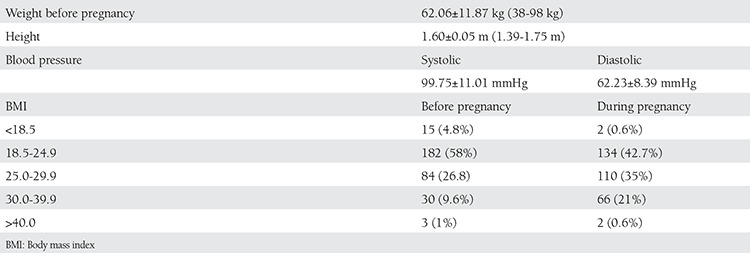
Descriptive clinical findings of the pregnant women
